# Swelling Behavior of Acrylate-Based Photoresist Polymers Containing Cycloaliphatic Groups of Various Sizes

**DOI:** 10.3390/ma17225465

**Published:** 2024-11-08

**Authors:** Choong-Jae Lee, Jinyoung Kim, Geon-Ho Lee, Jayoung Hyeon, Yura Choi, Namchul Cho

**Affiliations:** Department of Energy Engineering, Soonchunhyang University, 22 Soonchunhyang-ro, Asan 31538, Republic of Korea; cndwolee1397@sch.ac.kr (C.-J.L.); kjy1624@sch.ac.kr (J.K.); sinb0603@sch.ac.kr (G.-H.L.); jayoung@sch.ac.kr (J.H.); bnbn3238@sch.ac.kr (Y.C.)

**Keywords:** cycloaliphatic monomer, swelling ratio, molar volume, functional group, acrylate monomer

## Abstract

Photoresist polymers containing cycloaliphatic acrylic monomers have been synthesized for use in the microcircuits of semiconductors. Although cycloaliphatic acrylic monomers exhibit a high etch resistance and excellent thermal properties, their large size increases the distance between the main chains of the resulting polymers. This increased distance facilitates the penetration of a developer between the main chains, which leads to swelling and thus pattern collapse, distortion, and delamination, thereby complicating the fabrication of microcircuits. To solve this problem, various large developers were used in previous studies to reduce the swelling effect. However, these developers could not easily dissolve the unexposed regions of the resist. To overcome this issue, we designed photoresist polymers with smaller functional groups to decrease the degree of swelling. Specifically, ArF photoresist polymers were synthesized from monomers with various sizes of functional groups. We confirmed that the polymer synthesized using cyclohexyl methacrylate (CHMA), which had the smallest functional group, exhibited the shortest distance between the main chains. Consequently, this polymer showed the least swelling, with a swelling ratio of 109%. In contrast, the polymers synthesized using isobornyl acrylate (IBOA) and dicyclopentanyl methacrylate (TCDMA), which have large functional groups, exhibited greater distances between the main chains, resulting in swelling ratios of 114% and 112%, respectively. The polymer with a swelling ratio of 109% showed excellent patterning properties, while those with swelling ratios of 114% and 112% were delaminated by the developer. Our work introduces a novel approach to help reduce the swelling effect and achieve high-quality patterns in negative photoresists.

## 1. Introduction

Because of the increased demand for finer patterns, photolithography technology employing shorter-wavelength UV light that is applicable to narrower linewidths has been developed. In addition, diazonaphthoquinone (DNQ) and novolak-based resins capable of forming patterns under UV light irradiation have been extensively studied. The resulting patterns are classified into two categories, positive tone and negative tone, both of which are influenced by developer solutions, which dissolve photoresist polymers that contain hydroxyl groups or that remain uncrosslinked. To form positive-tone patterns, a photoresist film is dissolved by a developer upon exposure to UV light. In contrast, to form negative-tone patterns, unexposed regions of a photoresist film are dissolved by a developer. Positive photoresists employ a photoacid generator (PAG) to deprotect acid-labile groups in the polymer, thus altering the polarity of the photoresist polymer. In contrast, negative-tone photoresists utilize a crosslinker to crosslink the polymer chains and thus render the photoresist polymer nonpolar [1,2,3,4,5,6,7].

Nanometer-scale patterns have been fabricated using styrene-based and acrylic-based polymers with 248 nm and 193 nm light irradiation [1,8,9,10,11]. Unlike the photoresists prepared with styrene-based polymers, those prepared with acrylic-based polymers exhibited low etching resistance and low thermal stability. To solve this problem, cycloaliphatic groups, because of their high etch resistance and excellent thermal stability, have been incorporated into acrylic-based polymers [12,13,14,15,16,17]. However, because of the large size of these cycloaliphatic groups, alkaline developers can penetrate between the polymer chains during the developing process. This penetration causes swelling of the polymer, leading to pattern collapse, distortion, and delamination [18,19]. As the solubility of the polymer in the developer increases, the penetration of the developer into the polymer matrix increases, thus enhancing the swelling effect of the polymer. To solve this problem, crosslinking has been applied in previous studies to prevent the swelling of photoresist polymers during the developing process [20]. In addition, strategies to reduce the degree of penetration into the polymer using developers with higher molecular weight (M_w_) values have been studied [19]. However, the crosslinking strategy employed for conventional acrylic-based polymers is limited, as it does not involve the incorporation of cycloaliphatic groups. Consequently, polymers prepared via this approach suffer from low etching resistance and low thermal stability. Moreover, developers with high M_w_ values have significantly decreased solubilities. To overcome these limitations, Naito et al. proposed a strategy in which a PAG is employed to introduce crosslinking between the hydroxyl groups of conventional polymers with cycloaliphatic groups [21].

In this study, our objective was to identify a cycloaliphatic group that is not only thermally stable, but also effectively inhibits developer penetration. For this purpose, we synthesized polymers containing cycloaliphatic groups of various sizes. We used hydroxypropyl methacrylate (HPMA) as a crosslinking group and 5-oxotetrahydrofuran-3-yl methacrylate (GBLMA) as a polar group [22]. In addition, cyclohexyl methacrylate (CHMA), isobornyl acrylate (IBOA), and dicyclopentanyl methacrylate (TCDMA), which increased in size in the order of CHMA, TCDMA, IBOA, were used as cycloaliphatic groups. CHMA was relatively small, allowing shorter distances between the main chains of the resulting polymer compared to those in the other polymers. In contrast, IBOA and TCDMA were relatively large, leading to increased distances between the polymer main chains. Because of the shorter distances between their main chains, polymers containing CHMA effectively controlled the penetration of alkaline developers, such as tetramethyl ammonium hydroxide (TMAH). Thus, the unexposed regions of the resists were dissolved by the developer instead of the exposed regions. In contrast, because of the longer distances between their main chains, the polymers containing IBOA and TCDMA were penetrated by the alkaline developer. Thus, both the exposed and unexposed regions of the resists were dissolved by the developer. These results indicate that developer penetration can be effectively controlled by decreasing the size of the cycloaliphatic group.

## 2. Materials and Methods

### 2.1. Materials

Hydroxypropyl methacrylate (HPMA), propylene glycol monomethyl ether acetate (PGMEA), benzoyl peroxide (BPO), and tetramethyl ammonium hydroxide (TMAH) were purchased from Sigma-Aldrich (St. Louis, MO, USA). *N*-((Trifluoromethylsulfonyl)oxy)-5-norbornene-2,3-dicarboximide (NDI) was purchased from Alfa Aesar (Haverhill, MA, USA). Ethyl acetate (EA, 99.5%) was purchased from Samchun Chemical (Seoul, Republic of Korea). Acetone (99.5%) and n-hexane (95%) were purchased from Daejung Chemical (Siheung, Republic of Korea). Cyclohexyl methacrylate (CHMA), isobornyl acrylate (IBOA), dicyclopentanyl methacrylate (TCDMA), and 5-Oxotetrahydrofuran-3-yl methacrylate (GBLMA) were purchased from TCI (Tokyo, Japan).

### 2.2. Synthesis of an HPMA–GBLMA–CHMA (HGC) Copolymer

HGC copolymers were synthesized as follows. PGMEA (12.06 g) was added to a batch reactor at 85 °C. Then, HPMA (4.24 g), GBLMA (10 g), CHMA (9.87 g), and BPO (0.84 g) were dissolved in PGMEA (24.11 g) and stirred under ambient conditions. The resulting mixture was injected dropwise into the batch reactor over 3 h. The synthesis of the copolymer was complete after 16 h.

After the synthesis, precipitation was conducted as follows. The synthesized mixture was injected dropwise into hexane at a mixture–hexane volume ratio of 1:8, and the resulting solution was stirred for 1 h. The mixture was allowed to stand for 12 h until the copolymer was fully precipitated. Then, the hexane was removed. To obtain the desired polymer, the precipitant was dried for 12 h under vacuum.

The polymer was purified as follows. HGC (20 g) was dissolved in EA (200 mL) and the mixture was washed with deionized water (DIW, 1600 mL). This workup process was repeated three times. Then, the EA layer was evaporated under vacuum to yield the HGC copolymer.

### 2.3. Synthesis of an HPMA-GBLMA-IBOA (HGI) Copolymer

HGI copolymers were synthesized as follows. PGMEA (13.24 g) was added to a batch reactor at 85 °C. Then, HPMA (4.24 g), GBLMA (10 g), IBOA (12.25 g), and BPO (0.95 g) were dissolved in PGMEA (26.48 g) and stirred under ambient conditions. The resulting mixture was injected dropwise into the batch reactor over 3 h. The synthesis of the copolymer was complete after 16 h.

After the synthesis, precipitation was conducted as follows. The synthesized mixture was injected dropwise into hexane at a mixture–hexane volume ratio of 1:8, and the resulting solution was stirred for 1 h. The mixture was allowed to stand for 12 h until the copolymer was fully precipitated. Then, the hexane was removed. To obtain the desired polymer, the precipitant was dried for 12 h under vacuum.

The polymer was purified as follows. HGI (20 g) was dissolved in EA (200 mL) and the mixture was washed with DIW (1600 mL). This workup process was repeated three times. Then, the EA layer was evaporated under vacuum to yield the HGI copolymer.

### 2.4. Synthesis of an HPMA-GBLMA-TCDMA (HGT) Copolymer

HGT copolymers were synthesized as follows. PGMEA (13.59 g) was added to a batch reactor at 85 °C. Then, HPMA (4.24 g), GBLMA (10 g), TCDMA (12.95 g), and BPO (0.84 g) were dissolved in PGMEA (27.18 g) and stirred under ambient conditions. The resulting mixture was injected dropwise into the batch reactor over 3 h. The synthesis of the copolymer was complete after 16 h.

After the synthesis, precipitation was conducted as follows. The synthesized mixture was injected dropwise into hexane at a mixture–hexane volume ratio of 1:8, and the resulting solution was stirred for 1 h. The mixture was allowed to stand for 12 h until the copolymer was fully precipitated. Then, the hexane was removed. To obtain the desired polymer, the precipitant was dried for 12 h under vacuum.

The polymer was purified as follows. HGT (20 g) was dissolved in EA (200 mL) and the mixture was washed with DIW (1600 mL). This workup process was repeated three times. Then, the EA layer was evaporated under vacuum to yield the HGT copolymer.

### 2.5. Lithography Process

Photoresist resins were prepared as described previously by Asakura et al. [23] (PGMEA:PAG:HGC, HGI or HGT = 600:2:100, wt.%). The photoresist resins were coated on a silicon wafer (4 cm^2^) at 7000 rpm for 240 s. Then, the films were soft-baked at 90 °C for 1 min. Photomasks were aligned with the baked wafers and exposed to 365 nm UV light for 180 s. The HGC, HGI, and HGT polymers were then subjected to post-exposure baking (PEB) for 1 min at 120 °C, 90 °C, and 140 °C, respectively. Next, the wafers were dipped in the developer (TMAH and a mixture of acetone and hexane at a volume ratio of 4:6) for 15 min. Then, the HGC, HGI, and HGT polymer-based patterns were hard-baked at 170 °C, 140 °C, and 190 °C, respectively, for 60 s.

### 2.6. Determination of the Swelling Ratio

The degree of swelling of each polymer was calculated as the ratio of the weight change to the initial weight (Equation (1)), where W represents the weight of the swollen photoresist film and W_0_ represents the initial weight of the photoresist film. To measure the degree of swelling of the photoresist films, HGC, HGI, and HGT were spin-coated onto a wafer at 1000 rpm for 4 min. After the photoresist films were soft-baked at 90 °C, the weight of the coated photoresist films was measured. After 3 min of UV light exposure, the HGC and HGT photoresists were baked at their respective PEB temperatures for 1 min. After PEB, the photoresists were developed in TMAH for 1 min. Using the same procedure, additional HGC and HGT films were developed in an acetone/hexane cosolvent for 1 min. The developer remaining on the surface of the photoresists was blow-dried. Unlike the HGC and HGT photoresists, the HGI photoresist was a positive-tone photoresist [24]. Therefore, the coated and baked HGI photoresist was weighed and developed without UV exposure. The weight of the films was measured using an analytical balance (Ohaus, Pioneer^TM^ PX124M, Parsippany, NJ, USA).

(1) $${SR}_{W}=(W-W_{0})/W_{0}\times 100$$

### 2.7. Characterization

Proton nuclear magnetic resonance (^1^H NMR) measurements were conducted with a 400 MHz Bruker Ascend 400 spectrometer using dimethyl sulfoxide (DMSO)-d_6_. The M_w_ and polydispersity index (PDI) were measured by means of gel permeation chromatography (GPC, Waters, Milford, MA, USA) with tetrahydrofuran (THF) as the mobile phase at a flow rate of 1 mL min^−1^. KS-801, KS-802, and KS-803 columns were used for the GPC analyses. The glass transition temperatures (T_g_) of the polymers were determined by means of differential scanning calorimetry (DSC, Q20 from TA Instruments Ltd., New Castle, DE, USA) in the range of 30–300 °C at a heating rate of 5 °C per minute under a nitrogen atmosphere. The decomposition temperatures (T_d_) of the polymers were determined by means of thermogravimetric analysis (TGA, Q50 from TA Instruments Ltd., New Castle, DE, USA) in the range of 30–600 °C at a heating rate of 10 °C per minute under a nitrogen atmosphere. The thicknesses of the photoresist films were measured with a nonoptical profilometer (Alpha-Step, D-500, KLA, Milpitas, CA, USA). To determine the distance between the main chains of the polymers, a Rigaku Mini Flex diffractometer (Tokyo, Japan) operating with CuKα radiation was used to obtain X-ray diffraction (XRD) spectra in the 2*θ* range of 2–50°, and the d-spacing was calculated via Bragg’s equation (Equation (2)) from the *θ* value obtained from the XRD spectra.

(2) d=λ/2sinθ

## 3. Results and Discussion

### 3.1. Characterization of the Copolymers

**Figure 1:**
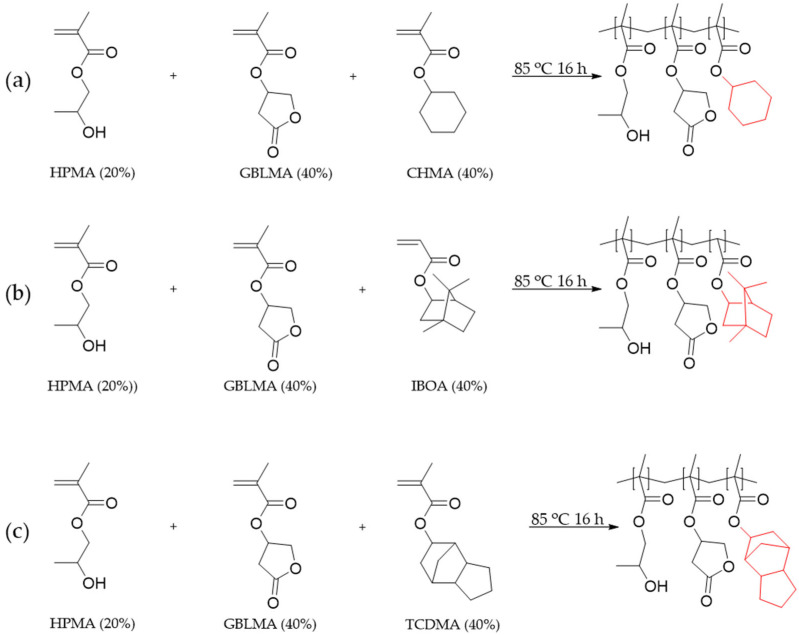
Design of acrylate-based polymers using free radical polymerization: (**a**) HGC (HPMA-GBLMA-CHMA), (**b**) HGI (HPMA-GBLMA-IBOA), and (**c**) HGT (HPMA-GBLMA-TCDMA).

To investigate the effect of different sizes of functional groups on ArF photoresist polymers, we synthesized three types of polymers with various cycloaliphatic functional groups (Figure 1). In the synthesized polymers, HPMA and GBLMA were employed as polar groups. CHMA, IBOA, and TCDMA, whose sizes decreased in the order of IBOA, TCDMA, CHMA, were utilized as cycloaliphatic groups. In this study, the polymer containing CHMA, IBOA, and TCDMA was named HGC (HPMA-GBLMA-CHMA), according to its composition; the polymer using IBOA was named HGI (HPMA-GBLMA-IBOA); and the polymer with TCDMA was named HGT (HPMA-GBLMA-TCDMA) in the same manner (Figure 1).

**Figure 2:**
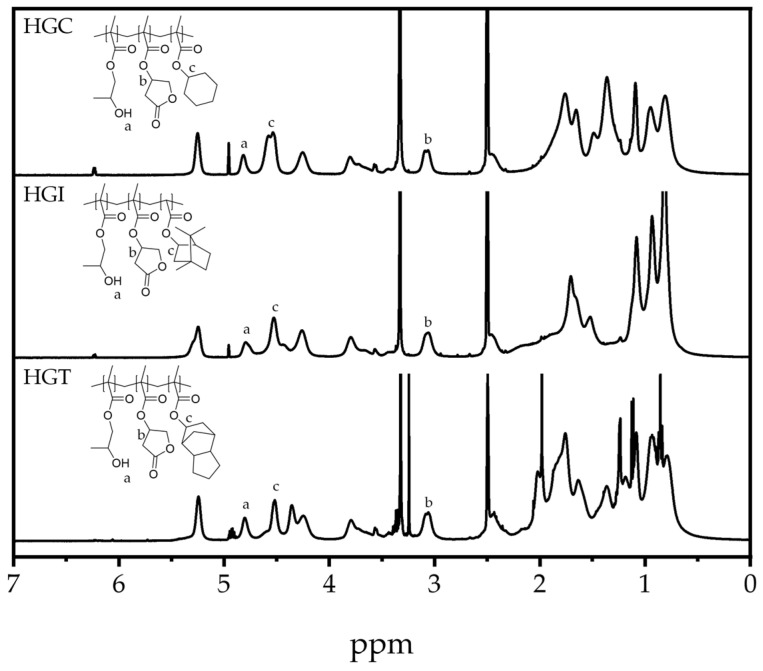
^1^H NMR spectra of the HGC, HGI, and HGT polymers in DMSO-d_6_.

The structures and compositions of the HGC, HGI, and HGT polymers were confirmed using ^1^H NMR, as shown in Figure 2. A peak attributed to the proton of the hydroxyl group in HPMA was observed at 4.81 ppm, while peaks attributed to the proton on the first carbon in the functional group of GBLMA were observed at 3.06 and 3.09 ppm as a doublet of doublets [25,26]. Peaks attributed to the proton on the first carbon in the functional group of CHMA were observed as a doublet at 4.54 and 4.58 ppm, a peak attributed to IBOA was observed as a singlet at 4.53 ppm, and a peak attributed to TCDMA was detected as a singlet at 4.52 ppm [27,28,29]. The compositions of each polymer were determined from the integrated areas of the specific peaks for each monomer. The polymer compositions shown in Table 1 varied with the feed ratios, which was attributed to the differences in the reactivity of each monomer [30,31].

**Figure 3:**
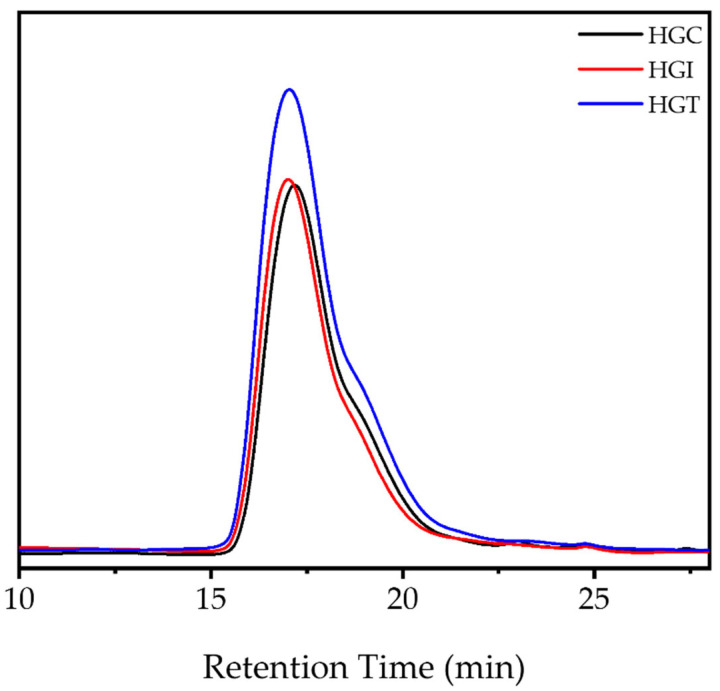
GPC data for the HGC, HGI, and HGT polymers.

The M_w_ of a polymer affects the penetration of a developer [32]. Liu et al. reported the effect of the polymer M_w_ on the patterning performance [28]. To investigate developer penetration, the M_w_ values of the polymers must be uniform [33,34]. In a previous study, Sohn et al. reported an M_w_ ratio of 0.75 [32]. The weight average molecular weight (M_w_) and polydispersity index (PDI) were determined through GPC (Figure 3, Table 1). The M_w_ values of the HGC, HGI, and HGT polymers were determined to be 18,712 g/mol, 20,205 g/mol, and 19,572 g/mol, respectively. In addition, the PDI values of the HGC, HGI, and HGT polymers were determined to be 2.44, 2.58, and 2.54, respectively. According to the GPC data, a similar M_w_ ratio (0.93) was observed for the synthesized polymers. Thus, we confirmed that differences in the M_w_ values did not affect the penetration of the developer.

**Figure 4:**
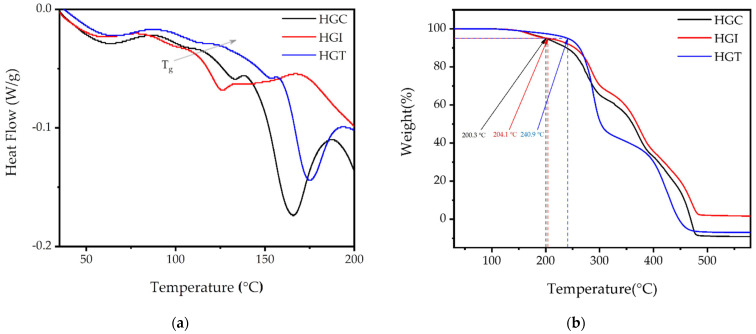
(**a**) DSC curves and (**b**) TGA curves of the HGC, HGI, and HGT polymers.

The T_g_ and T_d_ (5% weight loss temperature) values of a polymer affect the PEB temperature and the thermal stability of the polymer, respectively. The PEB temperature affects acid diffusion, thereby affecting the patterning results [35]. Thus, it is crucial to set an appropriate PEB temperature according to the polymer’s T_g_. The thermal properties of the HGC, HGI, and HGT polymers were evaluated using TGA and DSC. The T_g_ values were determined from the onset point of the steep endothermic heat flow after the first relaxation in the DSC curves, as described in a previous study [24]. This onset point was calculated from the intercept of two lines: one corresponding to the plateau region and the other corresponding to the steep endothermic transition region. In this way, the T_g_ values of the HGC, HGI, and HGT polymers were estimated to be 116 °C, 113 °C, and 132 °C, respectively (Figure 4a). These T_g_ values were similar to those previously reported for a copolymer with a similar structure [24,36,37]. Because of the tricycloalkyl group, HGC, HGI, and HGT were all thermally stable above 200 °C (Figure 4b). The PEB temperature of each polymer was set to 5 °C below the respective T_g_ value.

### 3.2. Evaluation of the Swelling Effect

**Scheme 1:**
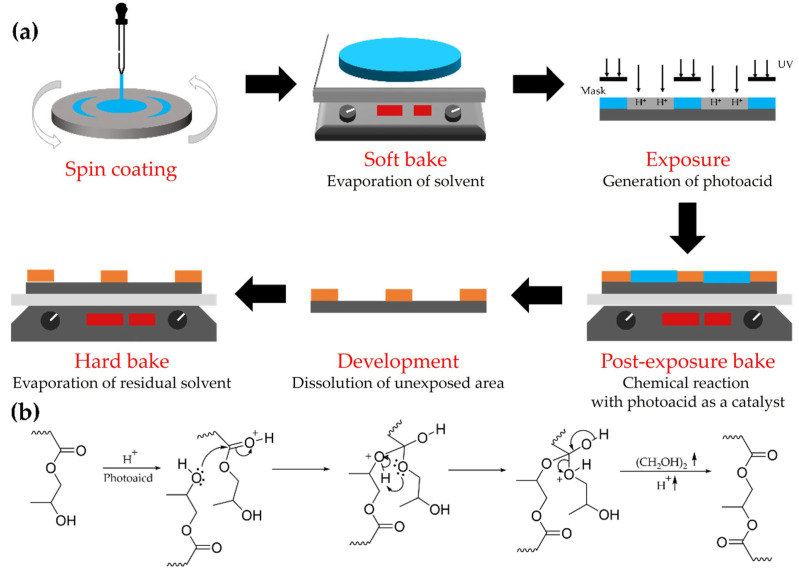
(**a**) Schematic of the photolithography procedure involving the photoresist and (**b**) the mechanism of polymer crosslinking for the photoresist via the exposure phase.

**Figure 5:**
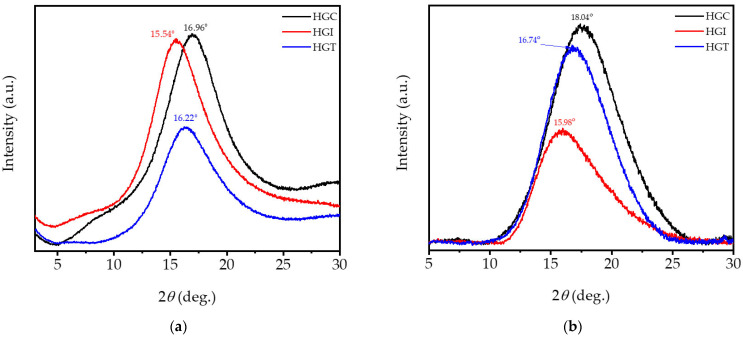
XRD spectra of the (**a**) unexposed and (**b**) exposed HGC, HGI, and HGT polymer-based photoresist films.

The polymers under investigation contained HPMA groups that underwent crosslinking when irradiated. As a result, the photoresist films incorporating these polymers displayed a negative tone following the development step [18]. After the exposure phase, the photoresist swelled because the developer penetrated into the crosslinked polymer matrix (Scheme 1). Thus, developer penetration into the photoresist film was affected by the distance between the main chains of the polymer [38]. An increase in the distance between the main chains of the polymer increased the penetration of the developer. Three polymers, CHMA, IBOA, and TCDMA, with various functional group sizes were used to control the d-spacing of the main chains (Equation (2)). The size of the functional group increased in the order of CHMA, TCDMA, IBOA. XRD measurements were conducted to confirm the distance between the main chains of the polymers. Before exposing the polymers to UV light, 2*θ* values of 16.96°, 15.54°, and 16.22° were observed for the HGC, HGI, and HGT photoresist films, respectively. In addition, the d-spacing values of the HGC, HGI, and HGT photoresist films before exposure to UV light were 5.22 Å, 5.70 Å, and 5.46 Å, respectively (Figure 5a, Table 2). A similar trend was observed after the exposure of the samples to UV light (Figure 5b, Table 2) [39,40], with 2*θ* values of 18.04°, 15.99°, and 16.74°, respectively, and d-spacing values of 4.91 Å, 5.54 Å, and 5.29 Å, respectively. The XRD data confirmed that the polymer containing CHMA, which had the smallest functional group, showed the shortest distance between its main chains. In contrast, the polymers containing IBOA and TCDMA, which have larger functional groups, showed greater distances between their main chains.

A photoresist film can be developed with various developers. A photoresist film exposed to a developer swells, which leads to pattern collapse, distortion, and delamination [13,15]. Atsushi et al. employed the quartz crystal microbalance (QCM) method to determine the degree of swelling by measuring the thickness of photoresist films [19]. Maura et al. determined the degree of swelling by measuring the weight change of photoresist films [41].

**Figure 6:**
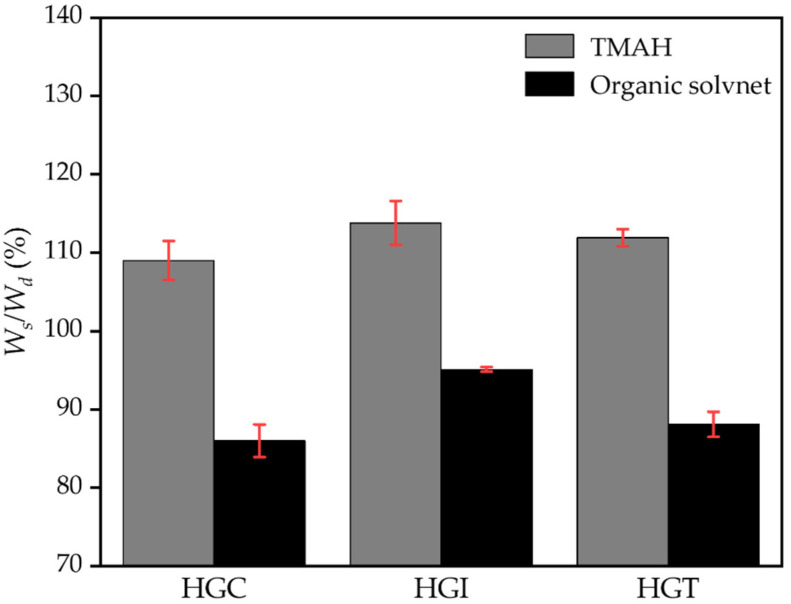
Swelling ratios calculated as the ratio of the weight change to the initial weight of the HGC, HGI, and HGT polymer-based photoresist films when developed with TMAH (gray) and acetone/hexane cosolvent (black). The error bars represent the standard deviations based on at least five specimens.

In this work, we examined how the degree of swelling varied between alkaline and acetone/hexane cosolvents. The degree of swelling was measured as described in Section 2.6. The measured swelling ratios indicated that when the alkaline developer TMAH was used, the weight increased in the order of HGC, HGT, HGI, with corresponding swelling ratios of 109%, 112%, and 114%, respectively (Figure 6). When the acetone/hexane cosolvent was used, the swelling ratios of HGC, HGI, and HGT were 86%, 95%, and 88%, respectively (Figure 6). When TMAH was used as the developer, the degree of swelling indicated that the HGI photoresist, which had the widest d-spacing, experienced the largest weight increase (Figure 5a and Figure 6). In contrast, the HGC photoresist, which had the smallest d-spacing, experienced the smallest weight increase (Figure 5a and Figure 6). These results indicated that the size of the functional group in the photoresist polymer affected the swelling caused by the developer.

The swelling of the photoresist films induced by the developer affected the film thickness (Figure S1 and Figure 2, Table 3). Specifically, when developed with TMAH, the photoresists underwent swelling, eventually resulting in complete dissolution of the films during prolonged development. Conversely, when organic solvents were employed, the films did not dissolve entirely; instead, the thickness decreased. In the case of HGI, photoacid induced the deprotection of the IBOA groups during the exposure phase, leading to an increased proportion of hydroxyl groups within the film. This chemical modification increased the polarity of the photoresist, which subsequently enhanced its solubility in organic solvents [42].

### 3.3. Lithographic Performance

**Figure 7:**
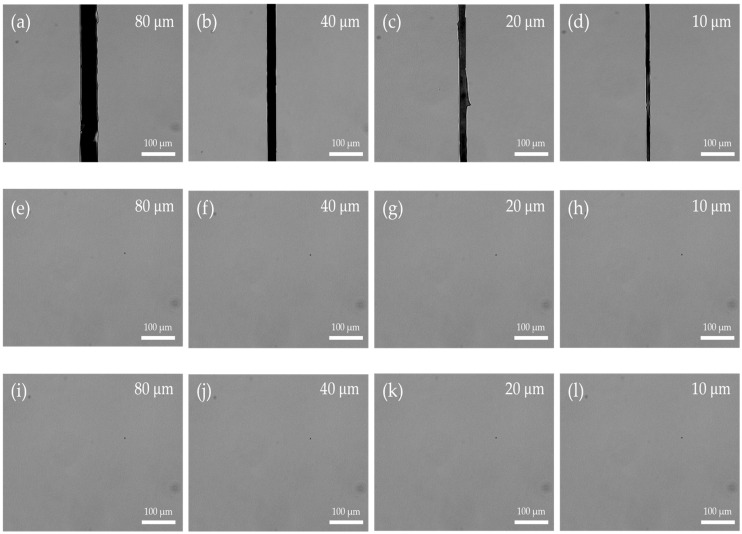
Optical microscopy (OM) images of the patterns of the (**a**–**d**) HGC, (**e**–**h**) HGI, and (**i**–**l**) HGT polymer-based photoresist films developed using TMAH depending on the line width (80, 40, 20, 10 μm).

**Figure 8:**
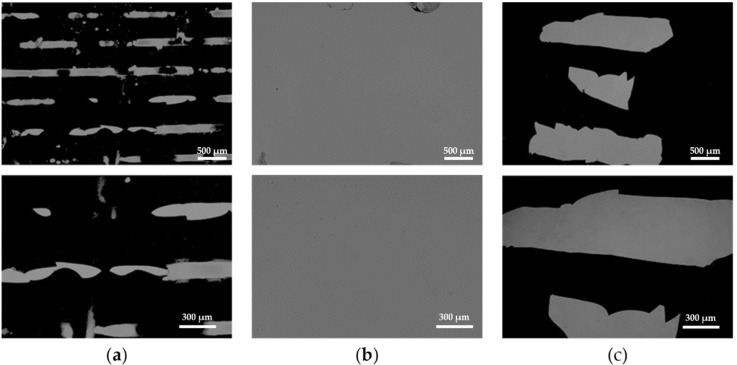
OM images of the transistor patterns of the HGC, HGI, and HGT polymer-based photoresist films developed using TMAH: (**a**) 5× (**top**) and 10× (**bottom**) magnification of the OM image of the transistor pattern of HGC, (**b**) 5× (**top**) and 10× (**bottom**) magnification of the OM image of the transistor pattern of HGI, and (**c**) 5× (**top**) and 10× (**bottom**) magnification of the OM image of the transistor pattern of HGT.

To investigate the effect of developer-induced swelling on photoresist films, two types of patterns were evaluated using HGC, HGI, and HGT photoresists. Each photoresist film was developed using TMAH. The line patterns of HGI and HGT, with swelling ratios of 112% and 114%, were completely delaminated after development (Figure 7e–l). In contrast, HGC, with a swelling ratio of 109%, retained its patterns even after development using TMAH (Figure 7a–d). The transistor patterns of the HGC films suffered from distortions and bridges (Figure 8a). Similarly, the line patterns of the HGI photoresist films were delaminated after the development of the films with TMAH (Figure 8b). The patterns of the HGT photoresist showed severe bridging and distortion (Figure 8c).

**Figure 9:**
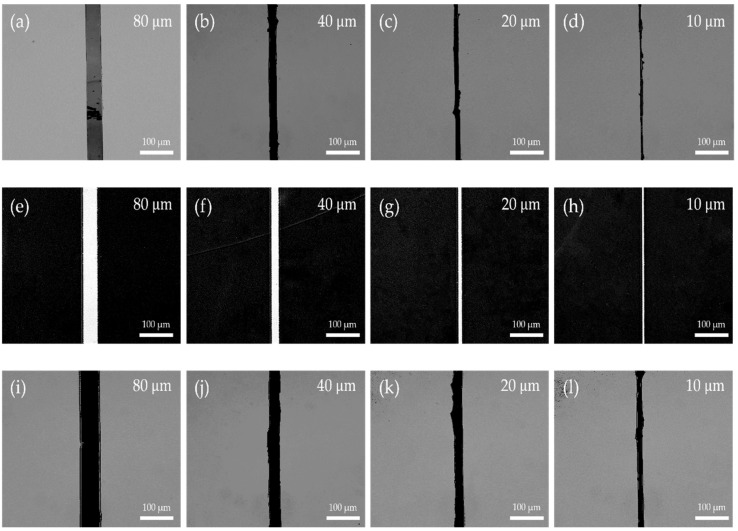
OM images of the patterns of the (**a**–**d**) HGC, (**e**–**h**) HGI, and (**i**–**l**) HGT polymer-based photoresist films developed using a mixture of acetone and hexane depending on the line width (80, 40, 20, 10 μm).

**Figure 10:**
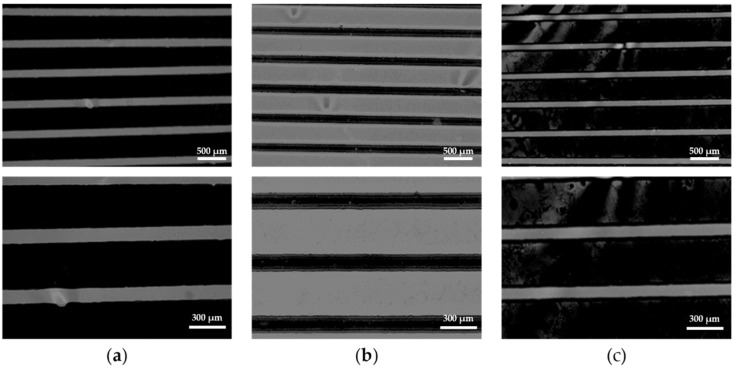
OM images of the transistor patterns of the HGC, HGI, and HGT polymer-based photoresist films developed in the acetone/hexane cosolvent: (**a**) 5× (**top**) and 10× (**bottom**) magnification of the OM image of the transistor pattern of HGC, (**b**) 5× (**top**) and 10× (**bottom**) magnification of the OM image of the transistor pattern of HGI, and (**c**) 5× (**top**) and 10× (**bottom**) magnification of the OM image of the transistor pattern of HGT.

When an acetone/hexane cosolvent, with larger molecules than TMAH, was used, all the photoresists exhibited good line patterns (Figure 9). Additionally, the HGC, HGI, and HGT photoresist films developed with the acetone/hexane cosolvent showed good transistor patterns (Figure 10). The HPMA in each polymer exhibited good solubility in acetone. Thus, a developer solution was prepared in hexane by adjusting the amount of acetone to control the polarity. In the HGC and HGT polymers, crosslinking was induced by HPMA in the exposed photoresist film, reducing the number of hydroxyl groups and making the photoresist film nonpolar [22]. As a result, the HGC and HGT polymer-based photoresist films formed negative-tone patterns. We also assumed that positive-tone patterns could be formed by the HGC and HGT photoresist films if nonpolar solvents, such as toluene and xylene, were used. In the nonpolar solvents, the HGC and HGT photoresist films rarely developed. In contrast, upon exposure of the HGI film, IBOA underwent deprotection via photoacid, increasing the proportion of hydroxyl groups. Consequently, the polarity of the photoresist film increased, leading to the formation of a positive-tone pattern upon development in the acetone/hexane cosolvent [42]. All the photoresist films were well-patterned upon development in the acetone/hexane cosolvent because the larger developer could not penetrate into the photoresist films [19]. We also evaluated the lithography performance with various acetone/hexane ratios (3:7, 4.5:55, 5:5, 6:4). The photoresist patterns were washed out when a higher proportion of acetone was used. In contrast, the photoresist patterns were not developed when a lower proportion of acetone was used. Therefore, it is important to utilize a developer with moderate polarity.

## 4. Conclusions

In this study, we investigated the degree of swelling induced by a developer depending on the size of functional groups. We synthesized three types of polymers, namely, HGC, HGI, and HGT, using HPMA with a polar hydroxyl group as a crosslinking monomer and GBLMA as a polar group. CHMA, IBOA, and TCDMA, with different sizes of functional groups, were employed as monomers. The swelling effect of the photoresist films was assessed by measuring the change in weight after development with TMAH. The results indicated that HGC, with the smallest d-spacing, exhibited the least swelling (109%), while HGI, with the largest d-spacing, showed the most significant swelling (114%). HGT exhibited a swelling ratio of 112%. The patterning results for all three polymers developed with TMAH were investigated. After development with TMAH, the line patterns of the HGI and HGT photoresists became delaminated or underwent severe distortion and bridging because of the higher swelling ratios, while the HGC photoresist retained its patterns with only slight distortion and bridging. In conclusion, we found that utilizing CHMA, which had the smallest size among the functional groups, effectively reduced the distance between the main chains of the polymers. This reduction in distance hindered the penetration of the developer, enabling the HGC photoresist polymer to retain its patterns. In contrast, IBOA and TCDMA, with larger functional groups, resulted in greater distances between the polymer main chains, which facilitated the penetration of the developers. These findings highlight the significant impact of controlling the size of functional groups to minimize swelling and achieve high-quality patterns. In addition, this study offers a promising direction for the development of advanced photoresist materials that meet the stringent demands of modern lithography processes.

## Figures and Tables

**Table 1:** Feed ratios and compositions of the HGC, HGI, and HGT polymers determined by ^1^H NMR and the average M_w_ and PDI determined by GPC.

Polymer	Molar Feed Ratios (Copolymer Composition)					M_w_ (g/mol)	PDI
	HPMA	GBLMA	CHMA	IBOA	TCDMA		
HGC	20 (15)	40 (28)	40 (57)	-	-	18,712	2.44
HGI	20 (17)	40 (31)	-	40 (52)	-	20,205	2.58
HGT	20 (21)	40 (37)	-	-	40 (42)	19,572	2.54

**Table 2:** Peaks in the XRD spectra of the unexposed (2*θ*^1^) and exposed (2*θ*^2^) photoresist films and the d-spacing values of the photoresist films with various polymers.

Polymer	2*θ*^1^ (°)	2*θ*^2^ (°)	d-Spacing at 2*θ*^1^ (Å)	d-Spacing at 2*θ*^2^ (Å)
HGC	16.96	18.04	5.22	4.91
HGI	15.54	15.99	5.70	5.54
HGT	16.22	16.74	5.46	5.29

**Table 3:** Thicknesses of the investigated polymer-based photoresist films measured by means of an Alpha-Step profilometer upon development with TMAH and an organic solvent (acetone/hexane cosolvent).

Polymer		Thickness (nm)	
		TMAH	Organic Solvent
HGC	Before	346	312
	After	-	244
HGI	Before	312	254
	After	-	-
HGT	Before	291	306
	After	-	118

## Data Availability

The original contributions presented in the study are included in the article/Supplementary Material, further inquiries can be directed to the corresponding author.

## Supplementary Materials

The following supporting information can be downloaded at: https://www.mdpi.com/article/10.3390/ma17225465/s1, Figure S1: Thickness measurement data by an Alpha-Step upon development process with acetone/hexane co-solvent of the investigated polymer-based photoresist films: (a,c,e) Undeveloped HGC, HGI, and HGT films; (b,d,f) developed HGC, HGI, and HGT films. Figure S2: Thickness measurement data by an Alpha-Step upon development process with TMAH of the investigated polymer-based photoresist films: (a–c) Undeveloped HGC, HGI, and HGT films.
