# Supplementary material for: Swelling Behavior of Acrylate-Based Photoresist Polymers Containing Cycloaliphatic Groups of Various Sizes
Source: Materials (Basel). 2024 Nov 8;17(22):5465. doi: 10.3390/ma17225465 (PMC11595884; doi:10.3390/ma17225465)
Supplement: Supplementary file 1 [file materials-17-05465-s001.zip › materials-3249086-supplementary.pdf]

materials MDPI

Supplementary Materials

Swelling Behavior of Acrylate-Based Photoresist Polymers Containing Cycloaliphatic Groups of Various Sizes

Choong-Jae Lee, Jinyoung Kim, Geon-Ho Lee, Jayoung Hyeon, Yura Choi and Namchul Cho *

Department of Energy Engineering, Soonchunhyang University, 22 Soonchunhyang-ro, Asan 31538, Republic of Korea; cndwolee1397@sch.ac.kr (C.-J.L.); kjy1624@sch.ac.kr (J.K.); sinb0603@sch.ac.kr (G.-H.L.); jayoung@sch.ac.kr (J.H.); bnbn3238@sch.ac.kr (Y.C.)

* Correspondence: chon7@sch.ac.kr

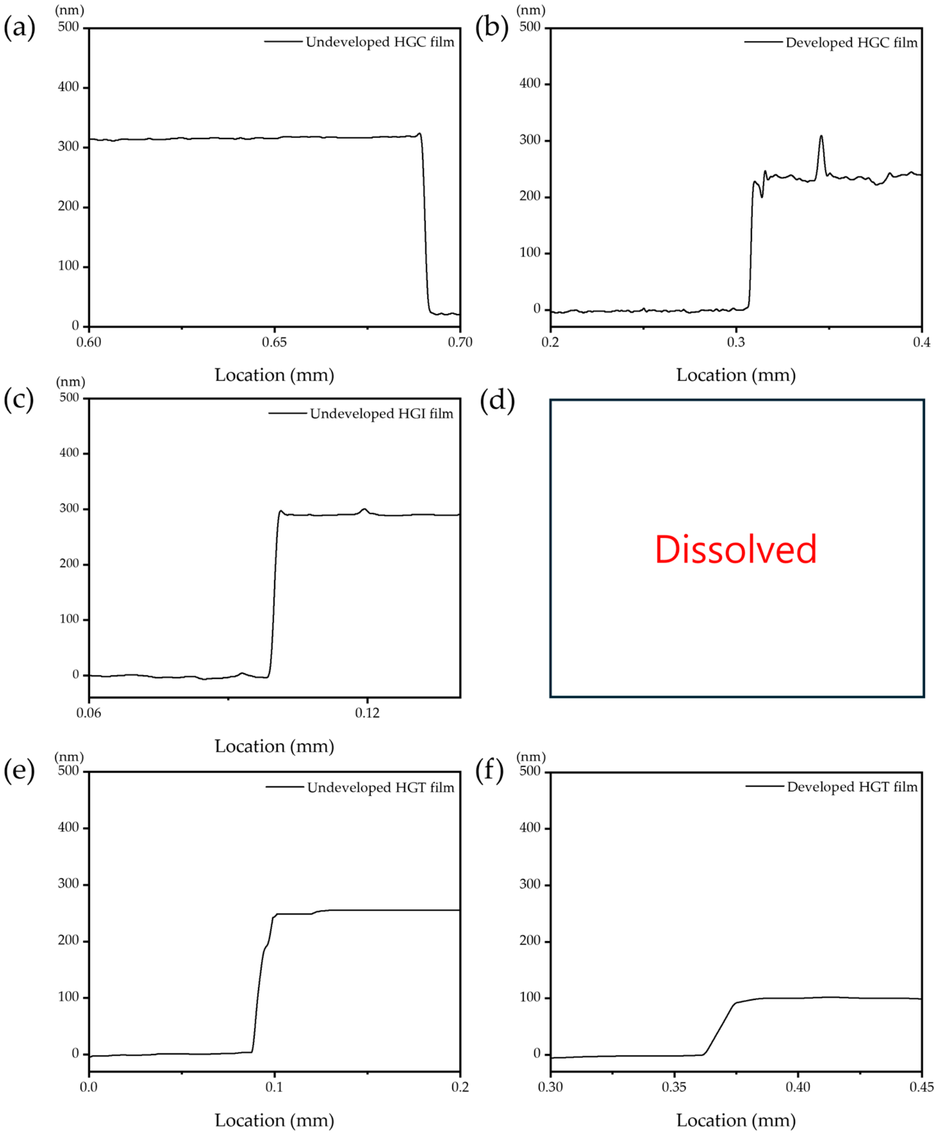

Figure S1. Thickness measurement data obtained by an Alpha-Step profilometer upon development process of the investigated polymer-based photoresist films with acetone/hexane co-solvent: (a, c, e) undeveloped HGC, HGI, and HGT films; (b, d, f) developed HGC, HGI, and HGT films.

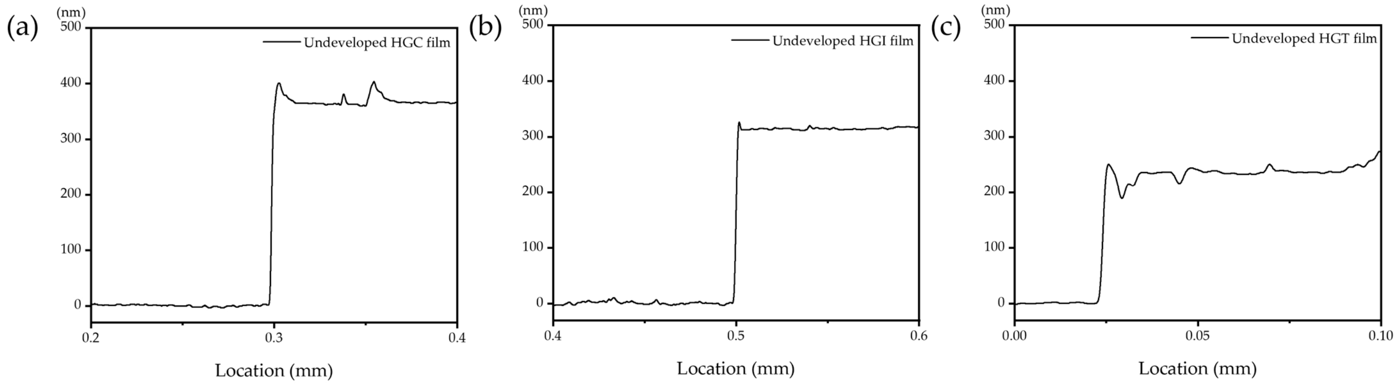

Figure S2. Thickness measurement data obtained by an Alpha-Step profilometer upon development process of the investigated polymer-based photoresist films with TMAH: (a-c) undeveloped HGC, HGI, and HGT films.
